# Nurse Practitioner Care, Scope of Practice, and End-of-Life Outcomes for Nursing Home Residents With Dementia

**DOI:** 10.1001/jamahealthforum.2024.0825

**Published:** 2024-05-10

**Authors:** Cyrus M. Kosar, Bishnu B. Thapa, Ulrike Muench, Christopher Santostefano, Emily A. Gadbois, Hyesung Oh, Pedro L. Gozalo, Momotazur Rahman, Elizabeth M. White

**Affiliations:** 1 Department of Health Services, Policy, and Practice, Brown University School of Public Health, Providence, Rhode Island; 2 Department of Social Behavioral Sciences, University of California at San Francisco School of Nursing, San Francisco

## Abstract

**Importance:**

Nursing home residents with Alzheimer disease and related dementias (ADRD) often receive burdensome care at the end of life. Nurse practitioners (NPs) provide an increasing share of primary care in nursing homes, but how NP care is associated with end-of-life outcomes for this population is unknown.

**Objectives:**

To examine the association of NP care with end-of-life outcomes for nursing home residents with ADRD and assess whether these associations differ according to state-level NP scope of practice regulations.

**Design, Setting, and Participants:**

This cohort study using fee-for-service Medicare claims included 334 618 US nursing home residents with ADRD who died between January 1, 2016, and December 31, 2018. Data were analyzed from April 6, 2015, to December 31, 2018.

**Exposures:**

Share of nursing home primary care visits by NPs, classified as minimal (<10% of visits), moderate (10%-50% of visits), and extensive (>50% of visits). State NP scope of practice regulations were classified as full vs restrictive in 2 domains: practice authority (authorization to practice and prescribe independently) and do-not-resuscitate (DNR) authority (authorization to sign DNR orders).

**Main Outcomes and Measures:**

Hospitalization within the last 30 days of life and death with hospice. Linear probability models with hospital referral region fixed effects controlling for resident characteristics, visit volume, and geographic factors were used to estimate whether the associations between NP care and outcomes varied across states with different scope of practice regulations.

**Results:**

Among 334 618 nursing home decedents (mean [SD] age at death, 86.6 [8.2] years; 69.3% female), 40.5% received minimal NP care, 21.4% received moderate NP care, and 38.0% received extensive NP care. Adjusted hospitalization rates were lower for residents with extensive NP care (31.6% [95% CI, 31.4%-31.9%]) vs minimal NP care (32.3% [95% CI, 32.1%-32.6%]), whereas adjusted hospice rates were higher for residents with extensive (55.6% [95% CI, 55.3%-55.9%]) vs minimal (53.6% [95% CI, 53.3%-53.8%]) NP care. However, there was significant variation by state scope of practice. For example, in full practice authority states, adjusted hospice rates were 2.88 percentage points higher (95% CI, 1.99-3.77; *P* < .001) for residents with extensive vs minimal NP care, but the difference between these same groups was 1.77 percentage points (95% CI, 1.32-2.23; *P* < .001) in restricted practice states. Hospitalization rates were 1.76 percentage points lower (95% CI, −2.52 to −1.00; *P* < .001) for decedents with extensive vs minimal NP care in full practice authority states, but the difference between these same groups in restricted practice states was only 0.43 percentage points (95% CI, −0.84 to −0.01; *P* < .04). Similar patterns were observed in analyses focused on DNR authority.

**Conclusions and Relevance:**

The findings of this cohort study suggest that NPs appear to be important care providers during the end-of-life period for many nursing home residents with ADRD and that regulations governing NP scope of practice may have implications for end-of-life hospitalizations and hospice use in this population.

## Key Points

**Question:**

Is nurse practitioner (NP) care associated with end-of-life outcomes for nursing home residents with Alzheimer disease and related dementias (ADRD), and do these associations differ between states with full vs restrictive NP scope of practice regulations?

**Findings:**

The results of this cohort study including 334 618 US nursing home residents with ADRD indicated that decedents with greater NP involvement at end of life had fewer hospitalizations and higher hospice use. The adjusted differences in outcomes between decedents with extensive vs minimal NP care were larger in states with full scope of practice regulations than in states with restrictive regulations.

**Meaning:**

These findings suggest that state regulations governing NP scope of practice may have implications for end-of-life hospitalizations and hospice use for nursing home residents with ADRD.

## Teaser

This cohort study examines whether there is an association between nurse practitioner care and end-of-life outcomes for nursing home residents with Alzheimer disease and related dementias and whether these associations differ by state-level nurse practitioner scope of practice regulations.

## Introduction

Nursing home residents living with Alzheimer disease and related dementias (ADRD) often experience unnecessary hospitalizations and other burdensome care in the last months of life.^1,2,3^ Better preemptive medical care in the nursing home—including timely recognition and treatment of acute change in condition, advance care planning, care coordination, and effective symptom management—can help avoid those difficult transitions.^4,5^ Timely access to hospice services can also help to facilitate comprehensive palliative care for individuals nearing the end of life.

An increase in ADRD-linked deaths, coupled with a rising shortage of geriatricians and other primary care physicians with geriatric expertise,^6,7,8^ has increased demand for clinicians with expertise in providing end-of-life care in nursing homes. Nurse practitioners (NPs) are playing an increasingly important role in the medical management of nursing home residents’ care^9,10,11^; however, the existing literature evaluating the effectiveness of NPs in nursing homes has a number of limitations.^12^ For example, existing studies are based on a small number of managed care models^13,14,15^ or rely on facility-level staffing measures that fail to capture individual clinician-patient encounters.^1,16,17^ Prior research has shown that NPs provide care coordination, palliative management, and advance care planning, all of which are critical elements of quality end-of-life care for individuals living with ADRD.^13,14,18^ However, no studies, to our knowledge, have empirically evaluated the association between NP care and end-of-life outcomes for this population.

State scope of practice regulations govern whether NPs can practice and prescribe without physician oversight (ie, full practice authority) or whether they must work under a collaborative practice agreement with a physician. Additionally, state regulations determine whether NPs are permitted to sign do-not-resuscitate (DNR) orders (ie, DNR authority). Thus, the ability of nursing home NPs to perform many of the functions necessary to treat acutely ill residents and provide end-of-life care, such as leading advanced care planning discussions or prescribing controlled medications for symptom relief, depends on the state in which the nursing home is located.^19,20,21^

The overarching goal of this study is to develop evidence that will inform workforce planning and policy efforts to provide high-quality end-of-life care to people with ADRD. Because NPs provide a significant amount of this care in nursing homes, it is important to assess how their practice, and policies regulating their practice, are associated with outcomes for this population. In this study, we first examined the association between the level of NP involvement in primary care and 2 end-of-life outcomes, hospitalizations and hospice use, among nursing home residents with ADRD who died between 2016 and 2018. We then examined whether these associations varied based on state NP scope of practice regulations governing practice authority and DNR authority.

## Methods

This cohort study was approved by the Brown University institutional review board, which granted a waiver for the requirement to obtain patient informed consent due to the use of deidentified claims data. This study followed the Strengthening the Reporting of Observational Studies in Epidemiology (STROBE) reporting guideline.

### Data Sources

We used 2015 to 2018 Medicare enrollment and claims data, including the Medicare Beneficiary Summary File (MBSF), Part A claims, and Part B Carrier claims file for 100% of beneficiaries with ADRD who died during the study window. We also used the Minimum Data Set to identify long-stay nursing home residents and obtained nursing home characteristics from the Certification and Survey Provider Enhanced Reports database (annual nursing home surveys) and Medicare Care Compare data on their website. Finally, we used the Area Health Resources File to obtain county characteristics and the zip code–level Social Deprivation Index developed by the Robert Graham Center.^22^ The Area Health Resources File contains over 1000 variables of combined data from Medicare and other government agencies on a diverse set of measures pertaining to county demographics, workforce, and health care supply, among other characteristics.^23^

### Study Population

The study population included individuals with ADRD who died between 2016 and 2018, were continuously enrolled in fee-for-service Medicare during the last year of life, and were long-stay nursing home residents. The study observation window included the last 9 months of life, with the first 8 months used as the baseline period to identify the cohort of nursing home residents, ascertain baseline resident characteristics, and assess NPs’ involvement in care. The last month of life was used to measure outcomes (eFigure 1 in Supplement 1). Individuals were classified as having ADRD if they had an active ADRD diagnosis indicated in the MBSF condition segment prior to the baseline period. Long-stay residence in a nursing home during the baseline period was assessed using the Residential History File algorithm,^24^ which links Medicare claims and assessment data to track daily health care utilization and site of care. Conventionally, individuals who reside in a nursing home for 100 or more days are considered long-stay residents; however, for our analysis, we required individuals to be in a nursing home for the entire 8 months prior to the last month of life, allowing for intermittent hospital stays.

### Measures

#### Nurse Practitioner Care

Our main explanatory variable was a 3-category variable representing the proportion of nursing home primary care visits provided by NPs in the 8 months prior to the last month of life. The measure excluded the last month of life, when outcomes were measured, to reduce endogeneity. Adapting methods from prior studies,^25,26^ we used provider specialty codes to identify carrier claims by primary care providers (ie, primary care clinicians), including NPs, physician assistants, and generalist physicians (ie, general practice, family practice, internal medicine, osteopathic medicine, geriatric medicine, and preventive medicine). We limited claims to encounters with BETOS (Berenson-Eggers type of service) code M4B (nursing home evaluation and management visits).

For each resident, we then calculated the percentage of primary care visits provided by NPs during the 8-month baseline period. We categorized this percentage as a 3-level measure: minimal involvement (<10% of visits conducted by NPs), moderate involvement (10%-50% of visits), and extensive involvement (>50% of visits). We chose this approach rather than assigning a main primary care provider for 3 reasons. First, our focus on the end-of-life period required limiting our observation window to a shorter period of time than other studies have used to assign primary care providers from claims data.^25,26^ Second, multiple providers are often involved in the end-of-life care of an individual. And third, Centers for Medicare & Medicaid Services regulations require all nursing home residents to have an attending physician who performs a minimum number of visits, meaning that an NP could never be the sole primary care provider in this setting.^27^ The distribution of the share of visits conducted by NPs across categories is shown in eFigure 2 in Supplement 1.

#### State Scope of Practice Regulations

We classified state scope of practice regulations based on the existing literature^28,29,30^ and a scoping review we conducted of state advance directive regulations. States were classified as having full practice authority if NPs were permitted to diagnose and treat patients without a collaborative or supervisory agreement with a physician and could prescribe schedule II through V medications without restriction.^28,29,30^ Otherwise, states were classified as restricted practice authority. States were classified as having full DNR authority if NPs were permitted to sign DNR orders, including Medical Orders for Life-Sustaining Treatment, and restricted DNR authority if NPs were not permitted to sign. Scope of practice regulations were classified as of 2016, the first year of the study, at which time 25 states had full practice authority and 32 had full DNR authority (eFigure 3 in Supplement 1).

#### Outcomes and Covariates

We derived 2 outcomes from inpatient and hospice claims: hospitalization within the last 30 days of life and hospice enrollment at death (ie, death occurred during an active hospice episode). Resident-level covariates included age at death, sex, race and ethnicity, dual Medicaid enrollment, indicators for 17 chronic conditions from the MBSF chronic condition segment, years since initial ADRD diagnosis (ie, the first date an *International Statistical Classification of Diseases and Related Health Problems, Tenth Revision*, diagnosis appeared on a claim), year of death, total number of primary care visits in the baseline period, and the share of visits from physician assistants. Race and ethnicity were included due to known racial and ethnic disparities in nursing home end-of-life care.^31^ Race and ethnicity data were obtained from the MBSF and included the self-reported categories of Asian, Black, Hispanic, North American Native, White, other, and unknown. We did not examine physician assistants as part of our primary exposure because they provided only a small fraction of overall visits and are subject to different scope of practice regulations than NPs. Geographic covariates included county population size and density, zip code Medicare Advantage penetration, and Social Deprivation Index.^22^

### Statistical Analysis

We first used linear probability models to estimate the overall association between NP care level and the 2 end-of-life outcomes, controlling for the aforementioned covariates. We then fit 2 additional models: 1 that additionally controlled for state practice authority and included an interaction between practice authority and NP care level and 1 that controlled for state DNR authority and included an interaction between DNR authority and NP care level. The significance of the interaction terms in these latter models indicated whether the associations between NP care and end-of-life outcomes differed for nursing home residents in states with restrictive vs full scope of practice regulations. Because practice patterns and patient characteristics in states with different regulations may vary in important ways, we included hospital referral region (HRR) fixed effects in all analyses. The HRRs are widely used geographic delineations of health care markets.^32^ There are 306 HRRs overall, of which about a third cross state boundaries. Thus, by including HRR fixed effects, the interaction term coefficients were derived from individuals in the same health care market but residing in different states with full vs restricted scope of practice regulations. Adjusted rates of outcomes were calculated via predictive margins, and robust standard errors were used in all analyses. Data were analyzed from April 6, 2015, to December 31, 2018, with Stata MP, version 17.0 (StataCorp LLC). A 2-side *P* < .05 was considered statistically significant.

We conducted 3 supplemental analyses. First, we examined sample characteristics by scope of practice regulation status, with or without HRR adjustment. Second, to better understand how residents’ health status varied by NP care level in states with restricted vs full scope of practice, we estimated the probability of hospitalization as a function of all study covariates, excluding the main explanatory variables (NP care and scope of practice). We then predicted the mean probability of hospitalization by NP care level in states with restricted vs full practice and DNR authority. Finally, we assessed the robustness of findings in analyses restricted to residents with a higher volume of baseline visits (ie, at least 8 visits during the 8-month baseline period or a mean of about 1 visit per month).

## Results

The sample included 334 618 nursing home decedents with ADRD (mean [SD] age at death, 86.6 [8.2] years; 69.3% female and 30.7% male; 10.1% Black, 1.7% Hispanic, 85.8% White, and 2.5% other race and ethnicity) (Table). Overall, 40.5% of residents received minimal NP care, 21.4% moderate NP care, and 38.0% extensive NP care. Compared with residents with minimal NP care, those with more NP involvement were more likely to be Black, to be Medicare and Medicaid dually enrolled, to be located in counties with larger populations, to have more baseline primary care visits, and to have a higher prevalence of anemia, chronic kidney disease, chronic obstructive pulmonary disease, heart failure, serious mental illness, and chronic wounds. eTable 1 in Supplement 1 shows the characteristics of residents in states with restricted vs full scope of practice, which were more comparable after HRR adjustment. eTable2 in Supplement 1 shows the predicted probability of hospitalization as a function of all covariates except for the 2 main explanatory variables (NP care and scope of practice) and shows how these predictions vary by NP care level in states with restricted vs full scope of practice. These results show that residents with moderate and extensive NP care had higher hospitalization risk, based on baseline characteristics, in both types of states.

**Table.** Characteristics of Nursing Home Decedents With Alzheimer Disease and Related Dementias, by Level of NP Care Received in the Months Before Death

Characteristic	Overall or pooled sample, No. (%) (N = 334 618)	Level of NP care for decedents, No. (%)^a^		
		Minimal (n = 135 540)	Moderate (n = 71 820)	Extensive (n = 127 258)
**Individual characteristics**				
Age at death, mean (SD), y	86.6 (8.2)	87.1 (8.2)	86.3 (8.3)	86.3 (8.3)
Sex				
Female	231 917 (69.3)	94 416 (69.7)	49 208 (68.5)	88 293 (69.4)
Male	102 701 (30.7)	41 124 (30.3)	22 612 (31.5)	38 965 (30.6)
Race and ethnicity				
Black	33 591 (10.1)	11 903 (8.8)	8107 (11.3)	13 581 (10.7)
Hispanic	5591 (1.7)	2939 (2.2)	1086 (1.5)	1566 (1.2)
White	286 475 (85.8)	115 585 (85.5)	61 018 (85.1)	109 872 (86.5)
Other^b^	8288 (2.5)	4830 (3.6)	1457 (2.0)	2001 (1.6)
Duration of dementia, mean (SD), y				
Dual Medicaid enrollment	5.3 (4.2)	5.4 (4.3)	5.3 (4.2)	5.2 (4.2)
Hypertension	251 773 (75.2)	100 630 (74.2)	54 765 (76.3)	96 378 (75.7)
Anemia	325 743 (97.3)	131 527 (97.0)	70 150 (97.7)	124 066 (97.5)
Atrial fibrillation	207 665 (62.1)	79 522 (58.7)	46 554 (64.8)	81 589 (64.1)
Cancer	125 788 (37.6)	49 862 (36.8)	27 696 (38.6)	48 230 (37.9)
Chronic kidney disease	70 042 (20.9)	28 268 (20.9)	15 405 (21.4)	26 369 (20.7)
COPD	234 774 (70.2)	92 525 (68.3)	51 414 (71.6)	90 835 (71.4)
Diabetes	99 857 (29.8)	38 820 (28.6)	22 417 (31.2)	38 620 (30.3)
Heart failure	193 982 (58.0)	77 444 (57.1)	42 994 (59.9)	73 544 (57.8)
Ischemic heart disease	176 360 (52.7)	69 385 (51.2)	38 616 (53.8)	68 359 (53.7)
Stroke or TIA	263 234 (78.7)	106 115 (78.3)	57 535 (80.1)	99 584 (78.3)
Drug use disorders	58 027 (17.3)	22 259 (16.4)	13 378 (18.6)	22 390 (17.6)
Peripheral vascular disease	5111 (1.5)	1745 (1.3)	1257 (1.8)	2109 (1.7)
Personality disorders	187 688 (56.1)	75 387 (55.6)	42 399 (59.0)	69 902 (54.9)
Pressure and chronic ulcers	12 309 (3.7)	3723 (2.7)	3136 (4.4)	5450 (4.3)
Bipolar disorder	108 606 (32.5)	40 258 (29.7)	26 299 (36.6)	42 049 (33.0)
Depression	37 210 (11.1)	11 626 (8.6)	9256 (12.9)	16 328 (12.8)
Schizophrenia	275 770 (82.4)	106 708 (78.7)	61 022 (85.0)	108 040 (84.9)
E/M visits during baseline, mean (SD), No.	19 071 (5.7)	6593 (4.9)	4578 (6.4)	7900 (6.2)
Share of E/M visits by PAs, mean (SD), %	12.7 (10.9)	9.5 (9.0)	14.6 (11.1)	15.0 (11.7)
**Residential characteristics**	5.1 (16.4)	7.8 (21.6)	6.9 (16.8)	1.3 (5.6)
Nursing home				
Star rating, mean (SD)				
Size, mean (SD), No. of beds	3.25 (1.33)	3.33 (1.31)	3.23 (1.34)	3.17 (1.34)
Beds Medicaid-financed, mean (SD), %	136.5 (78.4)	132.7 (83.5)	143.5 (82.7)	137.1 (69.9)
For-profit	61.0 (20.0)	60.6 (20.7)	61.2 (19.8)	61.5 (19.3)
State NP scope of practice regulations	222 815 (66.6)	86 778 (65.6)	49 351 (70.5)	86 686 (69.9)
Full practice authority				
Full do-not-resuscitate authority	63 331 (18.9)	25 745 (19.0)	13 144 (18.3)	24 442 (19.2)
County	169 228 (50.6)	69 813 (51.5)	37 406 (52.1)	62 009 (48.7)
Population size				
>1 000 000				
250 000 to 1 000 000	257 053 (76.8)	96 706 (71.4)	58 309 (81.2)	102 038 (80.2)
<250 000	51 768 (15.5)	24 780 (18.3)	9554 (13.3)	17 434 (13.7)
Population (1000s) per square mile, mean (SD), No.	25 793 (7.7)	14 050 (10.4)	3957 (5.5)	7786 (6.1)
Zip code	1.6 (5.2)	1.8 (6.2)	1.9 (5.9)	1.2 (3.5)
Medicare Advantage penetration rate, mean (SD)				
Social Deprivation Index, mean (SD)	30.0 (10.0)	30.0 (10.0)	30.0 (10.0)	30.0 (10.0)
	47.6 (27.4)	48.7 (27.1)	46.8 (27.9)	46.8 (27.5)

Abbreviations: COPD, chronic obstructive pulmonary disease; E/M, evaluation and management; NP, nurse practitioner; TIA, transient ischemic attack; PA, physician assistant.

^a^ Individuals were classified based on the percentage of primary care E/M visits conducted by NPs during the baseline period, ie, minimal NP care (<10% of visits), moderate NP care (10%-50% of visits), and extensive NP care (>50% of visits).

^b^ Includes Asian, North American Native, other race, and unknown race as measured in the Master Beneficiary Summary File.

**Figure 1.**
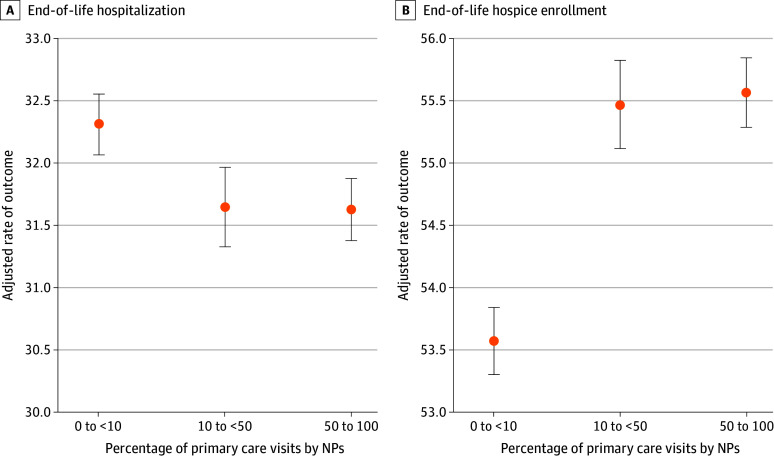
Association Between Nurse Practitioner (NP) Care and End-of-Life Outcomes Among Nursing Home Residents With Alzheimer Disease and Related Dementias End-of-life hospitalizations defined as those occurring during the last 30 days of life; end-of-life hospice enrollment defined as being enrolled in hospice at the time of death. Error bars represent 95% CI.

Figure 1 shows adjusted mean rates of hospitalization within the last 30 days of life and death with hospice for nursing home residents by NP care level (eTable 3 in Supplement 1 shows the point estimates). Adjusted hospitalization rates were lower for residents who had moderate NP care (31.6% [95% CI, 31.3%-32.0%]) and extensive NP care (31.6% [95% CI, 31.4%-31.9%]) compared with residents with minimal NP care (32.3% [95% CI, 32.1%-32.6%]). Adjusted hospice rates were higher for residents who had moderate NP care (55.5% [95% CI, 55.1%-55.8%]) and extensive NP care (55.6% [95% CI, 55.3%-55.9%]) compared with residents with minimal NP care (53.6% [95% CI, 53.3%-53.8%]).

**Figure 2.**
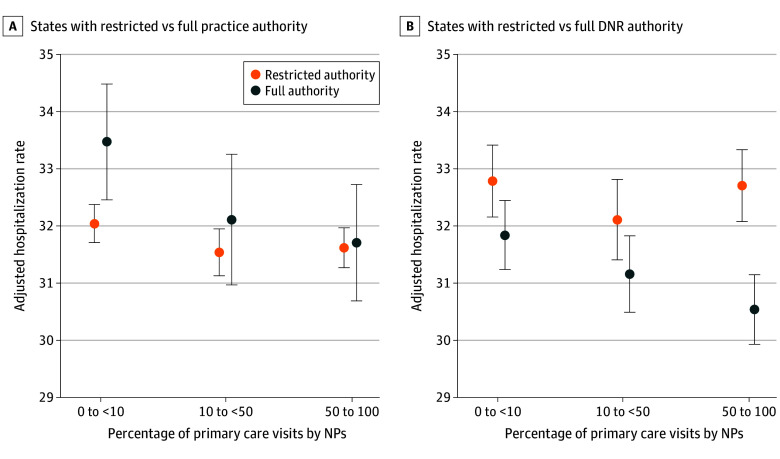
Association Between Nurse Practitioner (NP) Care and End-of-Life Hospitalization in States With Restricted vs Full Practice Authority and Do-Not-Resuscitate (DNR) Authority End-of-life hospitalizations defined as those occurring during the last 30 days of life.

We observed that the associations between NP care and hospitalization varied by state scope of practice. Figure 2 shows adjusted hospitalization rates by NP care level in states with restricted vs full practice authority and restricted vs full DNR authority. eTable 4 and eTable 5 in Supplement 1 show the corresponding point estimates. In states with restricted practice authority, compared with an adjusted mean hospitalization rate of 32.0% for residents with minimal NP care, hospitalization rates were 0.51 percentage points lower (95% CI, −0.96 to −0.05; *P* = .03) for residents with moderate NP care and 0.43 percentage points lower (95% CI, −0.84 to −0.01; *P* = .04) for residents with extensive NP care (eTable 4 in Supplement 1). The difference between the extensive vs minimal NP group was larger in full practice authority states (−1.34 [95% CI, −2.19 to −0.49]; interaction *P* = .002) (eTable 5 in Supplement 1). Compared with an adjusted mean hospitalization rate of 33.5% for residents with minimal NP care, hospitalization rates were 1.36 percentage points lower (95% CI, −2.28 to −0.45; *P* = .004 for residents with moderate NP care and 1.76 percentage points lower (95% CI, −2.52 to −1.00; *P* < .001) for residents with extensive NP care (eTable 4 in Supplement 1). Hospitalization rates across NP care levels followed similar patterns in states with full vs restrictive DNR authority (Figure 2). One distinction was that the DNR authority main effect coefficient was not statistically significant, whereas the practice authority main effect was positive and statistically significant (1.43 [95% CI, 0.22-2.65]; *P* = .02) (eTable 5 in Supplement 1). This means that mean hospitalization rates for residents of full practice authority states were higher than rates for residents of restricted practice authority states but converged at increasing levels of NP care.

**Figure 3.**
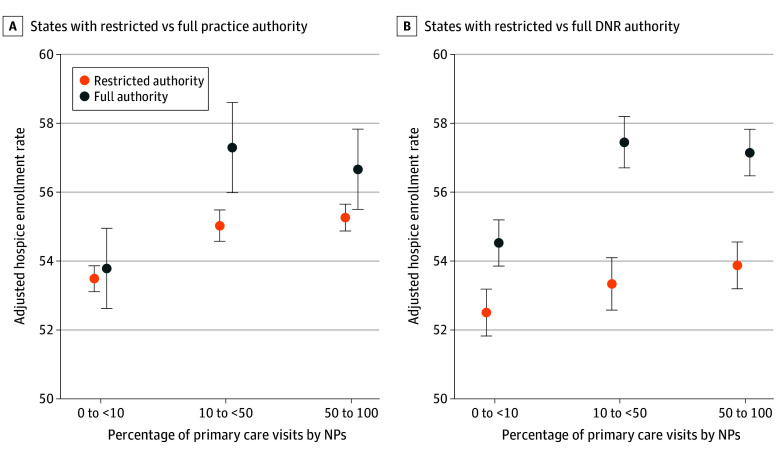
Association Between Nurse Practitioner (NP) Care and End-of-Life Hospice Enrollment in States With Restricted vs Full Practice Authority and Do-Not-Resuscitate (DNR) Authority End-of-life hospice enrollment defined as being enrolled in hospice at the time of death.

We similarly observed that the associations between NP care and hospice use varied by state scope of practice. Figure 3 shows adjusted hospice rates by NP care level in states with restricted vs full practice authority and restricted vs full DNR authority. eTable 6 and eTable 7 in Supplement 1 show the corresponding point estimates. In states with restricted practice authority, compared with adjusted hospice rates of 53.5% for residents with minimal NP care, hospice rates were 1.53 percentage points higher for residents with moderate NP care (95% CI, 1.04-2.03; *P* < .001) and 1.77 percentage points higher for residents with extensive NP care (95% CI, 1.32-2.23; *P* < .001) (eTable 6 in Supplement 1). The differences across groups were larger in states with full practice authority. Compared with adjusted hospice rates of 53.8% for residents with minimal NP care, rates for residents with moderate NP care were 3.51 percentage points higher (95% CI, 2.45-4.56; *P* < .001), and rates for residents with extensive NP care were 2.88 percentage points higher (95% CI, 1.99-3.77; *P* < .001) (eTable 6 in Supplement 1). These findings reflect statistically significant interactions between NP care and practice authority, both with regard to moderate vs minimal NP care (1.97 [95% CI, 0.81-3.14]; interaction *P* = .001) and to extensive vs minimal NP care (1.11 [95% CI, 0.12-2.09]; interaction *P* = .03) (eTable 7 in Supplement 1). Hospice rates across NP care levels followed similar patterns in full vs restrictive DNR authority states (Figure 3). However, mean hospice rates, without respect to NP care level, were still higher in full vs restricted DNR authority states, as indicated by the statistically significant DNR authority main effect coefficient (2.02 [95% CI, 0.78-3.23]; *P* = .001) (eTable 7 in Supplement 1). Findings were broadly consistent in analyses restricted to residents with a higher volume of baseline visits (eTable 8 and eTable 9 in Supplement 1).

## Discussion

In this cohort study assessing a national sample of nursing home decedents with ADRD enrolled in fee-for-service Medicare, over one-third of residents (38%) received the majority of their primary care from NPs in the months before death. This is consistent with prior studies showing that NPs are providing an increasing proportion of medical care in nursing homes.^10,11^ Given the growing size of the ADRD population and shortages of geriatricians and other primary care physicians with the skills and capacity to see patients in nursing homes,^8^ these findings suggest that NPs may be filling an important care role during the end-of-life period for many nursing home residents with ADRD.

To our knowledge, this is the first empirical examination of the association between NP care and end-of-life outcomes. Controlling for patient characteristics, visit volume, and geographic factors, we observed that decedents who received moderate and extensive NP care had modestly lower hospitalization rates in the last 30 days of life and higher rates of hospice use compared with decedents with no or minimal exposure to NPs. Decedents with more NP care received more primary care visits on average; however, our models controlled for visit volume, and our findings remained robust when we limited analyses to patients who received a higher volume of visits. This finding suggests that the outcomes are not just associated with NPs providing more frequent visits and may reflect NPs performing specific roles that may help preempt hospitalizations and increase hospice referrals for people with ADRD nearing the end of life. For example, an increasing number of nursing homes are employing or contracting with medical practices that employ NPs to provide more consistent in-house medical coverage.^9^ In these roles, NPs can manage acute and chronic illness, coordinate care, provide palliative care, facilitate advanced care planning, communicate with families, and mentor bedside nursing staff,^13,14,18^ all of which may contribute to improved end-of-life outcomes.

Notably, the association between NP care and end-of-life outcomes in the present study varied based on state scope of practice regulations. We found that the differences in outcomes between residents receiving minimal NP care and residents receiving moderate or extensive NP care were larger in states with full authority regulations than in states with restricted authority regulations. We know from the literature that less restrictive NP scope of practice regulations appear to be associated with expanded health care access, particularly for rural and vulnerable populations.^21,33^ Our findings indicating associations between NP scope of practice regulations and patient outcomes are an important new contribution to this literature.

There are a number of ways in which NP scope of practice regulations may influence care patterns. Full DNR authority may encourage NPs to be more proactive about initiating goals of care discussions with patients and families since NPs can sign a DNR order at the time of discussion, when patients and families agree, rather than having to find a physician to sign the order, which requires extra time and can result in delays in the order being executed. Timely completion of these orders is particularly important when a patient is acutely decompensating to prevent an unnecessary escalation in care due to a physician being unavailable to sign the DNR order. Practice authority, particularly the ability to prescribe schedule II through V medications, may affect access to medications typically used in palliative and end-of-life care to provide pain and symptom relief. Our data are from 2015 through 2018, before many states adopted electronic prescribing for controlled substances and instead required paper prescriptions. Thus, in nursing homes in which physicians are physically present for only a few hours a week or less but an NP is present more regularly, there could potentially be delays or gaps in access to palliative medications if NPs can only prescribe a limited supply or duration of medication under state law. Additionally, while the Centers for Medicare & Medicaid Services does not allow NPs to certify patients as terminally ill to receive hospice services under Medicare, NPs may still refer patients and serve as the attending physician under hospice.^27^ In states with restricted practice authority, it may be more difficult for NPs to serve in that role and order treatments requested by the hospice agency. While we examined practice authority and DNR authority separately in our analyses because we expected that these different regulations could be associated with care via different mechanisms, it may also be the case that these different regulations may have an additive association. This is an area for further exploration.

### Limitations

This study has certain limitations. First, we are unable to draw conclusions as to causal relationships between our variables of interest. While the inclusion of a range of covariates and HRR fixed effects strengthens the estimation strategy, residual confounding is still a concern in this observational study. Second, our analyses were focused only on nursing home residents with ADRD enrolled in fee-for-service Medicare and cannot be generalized further. The use of HRR fixed effects also reduced generalizability since estimates do not rely on any variation in outcomes from areas that do not cross state boundaries. Third, our use of administrative data limits our ability to measure dementia severity, and measurement error may differ across levels of NP care. Fourth, because provider specialty codes in carrier claims do not disaggregate NP specialty, it is possible that we captured some NPs working in specialty roles, such as psychiatry or palliative care, rather than in primary care. Similarly, by limiting the analysis to generalist physician codes, we may have missed some specialist physicians who served as primary attending physicians in a nursing home. Finally, we were unable to classify practice models or identify specific mechanisms influencing end-of-life outcomes, such as the initiation of advance care planning due to the use of administrative data.

## Conclusions

The findings of this cohort study suggest that NPs are important care providers during the end-of-life period for many nursing home residents with ADRD. The findings also suggest that state regulations governing whether NPs can practice without physician supervision and sign DNR orders may have implications for end-of-life hospitalizations and hospice use in this population.

## Notes

Supplement 1. **eFigure 1.** Study Timeline **eFigure 2.** Distribution of the Share of Primary Care Evaluation and Management Visits Conducted by Nurse Practitioners **eFigure 3.** Scope-of-Practice Regulations by State at the Time of Study **eTable 1.** Crude and Adjusted Characteristics of Individuals in States With Restricted vs Full Practice and Do Not Resuscitate Order Authority for Nurse Practitioners **eTable 2.** Predicted Probability of End-of-Life Hospitalization by Level of Nurse Practitioner Care and Scope-of-Practice Regulation **eTable 3.** Association Between Nurse Practitioner Care Level and End-of-life Outcomes Among Nursing Home Residents With Dementia **eTable 4.** Adjusted End-of-Life Hospitalization Rates in States With Restricted vs Full Practice and Do Not Resuscitate Order Authority for Nurse Practitioners **eTable 5.** Association Between Nurse Practitioner Care Level, Scope-of-Practice Regulation, and their Interaction With End-of-life Hospitalizations **eTable 6.** Adjusted End-of-Life Hospice Enrollment Rates in States With Restricted vs Full Practice and Do Not Resuscitate Order Authority for Nurse Practitioners **eTable 7.** Association Between Nurse Practitioner Care Level, Scope-of-Practice Regulation, and Their Interaction With End-of-life Hospice Enrollment **eTable 8.** Adjusted End-of-Life Hospitalization Rates by Nurse Practitioner Level Care Level and Scope-of-Practice Regulation Among Residents With High Visit Volume **eTable 9.** Adjusted End-of-Life Hospice Enrollment Rates by Nurse Practitioner Care Level and Scope-of-Practice Regulation among Residents with High Visit Volume

Supplement 2.
Data Sharing Statement
