# Supplementary material for: Nurse Practitioner Care, Scope of Practice, and End-of-Life Outcomes for Nursing Home Residents With Dementia
Source: JAMA Health Forum. 2024 May 10;5(5):e240825. doi: 10.1001/jamahealthforum.2024.0825 (PMC11087831; doi:10.1001/jamahealthforum.2024.0825)
Supplement: Supplement 1. — eFigure 1. Study Timeline eFigure 2. Distribution of the Share of Primary Care Evaluation and Management Visits Conducted by Nurse Practitioners eFigure 3. Scope-of-Practice Regulations by State at the Time of Study eTable 1. Crude and Adjusted Characteristics of Individuals in States With Restricted vs Full Practice and Do Not Resuscitate Order Authority for Nurse Practitioners eTable 2. Predicted Probability of End-of-Life Hospitalization by Level of Nurse Practitioner Care and Scope-of-Practice Regulation eTable 3. Association Between Nurse Practitioner Care Level and End-of-life Outcomes Among Nursing Home Residents With Dementia eTable 4. Adjusted End-of-Life Hospitalization Rates in States With Restricted vs Full Practice and Do Not Resuscitate Order Authority for Nurse Practitioners eTable 5. Association Between Nurse Practitioner Care Level, Scope-of-Practice Regulation, and their Interaction With End-of-life Hospitalizations eTable 6. Adjusted End-of-Life Hospice Enrollment Rates in States With Restricted vs Full Practice and Do Not Resuscitate Order Authority for Nurse Practitioners eTable 7. Association Between Nurse Practitioner Care Level, Scope-of-Practice Regulation, and Their Interaction With End-of-life Hospice Enrollment eTable 8. Adjusted End-of-Life Hospitalization Rates by Nurse Practitioner Level Care Level and Scope-of-Practice Regulation Among Residents With High Visit Volume eTable 9. Adjusted End-of-Life Hospice Enrollment Rates by Nurse Practitioner Care Level and Scope-of-Practice Regulation among Residents with High Visit Volume [file jamahealthforum-e240825-s001.pdf]

Supplementary Online Content

Kosar CM, Thapa BB, Muench U, et al. Nurse practitioner care, scope of practice, and end-of-life outcomes for nursing home residents with dementia. *JAMA Health Forum*. 2024;5(5):e240825. doi:10.1001/jamahealthforum.2024.0825

eFigure 1. Study Timeline

eFigure 2. Distribution of the Share of Primary Care Evaluation and Management Visits Conducted by Nurse Practitioners

eFigure 3. Scope of Practice Regulations by State at the Time of Study

eTable 1. Crude and Adjusted Characteristics of Individuals in States with Restricted vs Full Practice and Do Not Resuscitate Order Authority for Nurse Practitioners

eTable 2. Predicted Probability of End-of-Life Hospitalization by Level of Nurse Practitioner Care and Scope of Practice Regulation

eTable 3. Association Between Nurse Practitioner Care Level and End-of-life Outcomes Among Nursing Home Residents with Dementia

eTable 4. Adjusted End-of-Life Hospitalization Rates in States with Restricted vs Full Practice and Do Not Resuscitate Order Authority for Nurse Practitioners

eTable 5. Association Between Nurse Practitioner Care Level, Scope of Practice Regulation, and their Interaction with End-of-life Hospitalizations

eTable 6. Adjusted End-of-Life Hospice Enrollment Rates in States with Restricted vs Full Practice and Do Not Resuscitate Order Authority for Nurse Practitioners

eTable 7. Association Between Nurse Practitioner Care Level, Scope of Practice Regulation, and Their Interaction with End-of-life Hospice Enrollment

eTable 8. Adjusted End-of-Life Hospitalization Rates by Nurse Practitioner Level Care Level and Scope of Practice Regulation Among Residents with High Visit Volume

eTable 9. Adjusted End-of-Life Hospice Enrollment Rates by Nurse Practitioner Care Level and Scope of Practice Regulation among Residents with High Visit Volume

This supplementary material has been provided by the authors to give readers additional information about their work.

© 2024 Kosar CM et al. *JAMA Health Forum*.

e**Figure 1.** Study Timeline

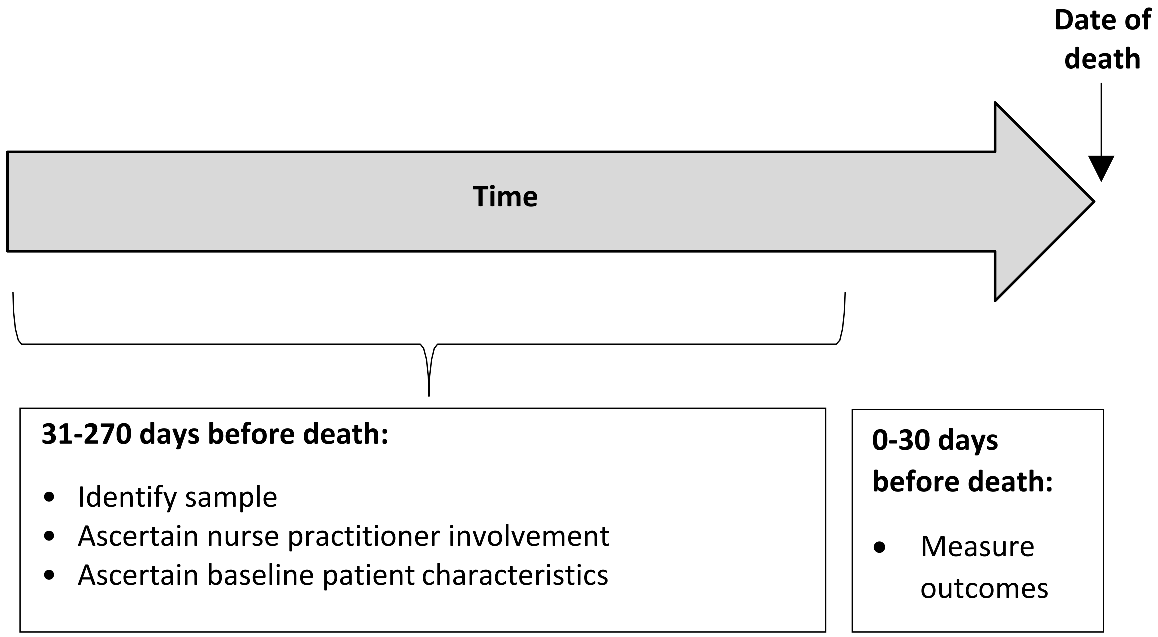

© 2024 Kosar CM et al. *JAMA Health Forum*.

eFigure 2. Distribution of the Share of Primary Care Evaluation and Management Visits Conducted by Nurse Practitioners

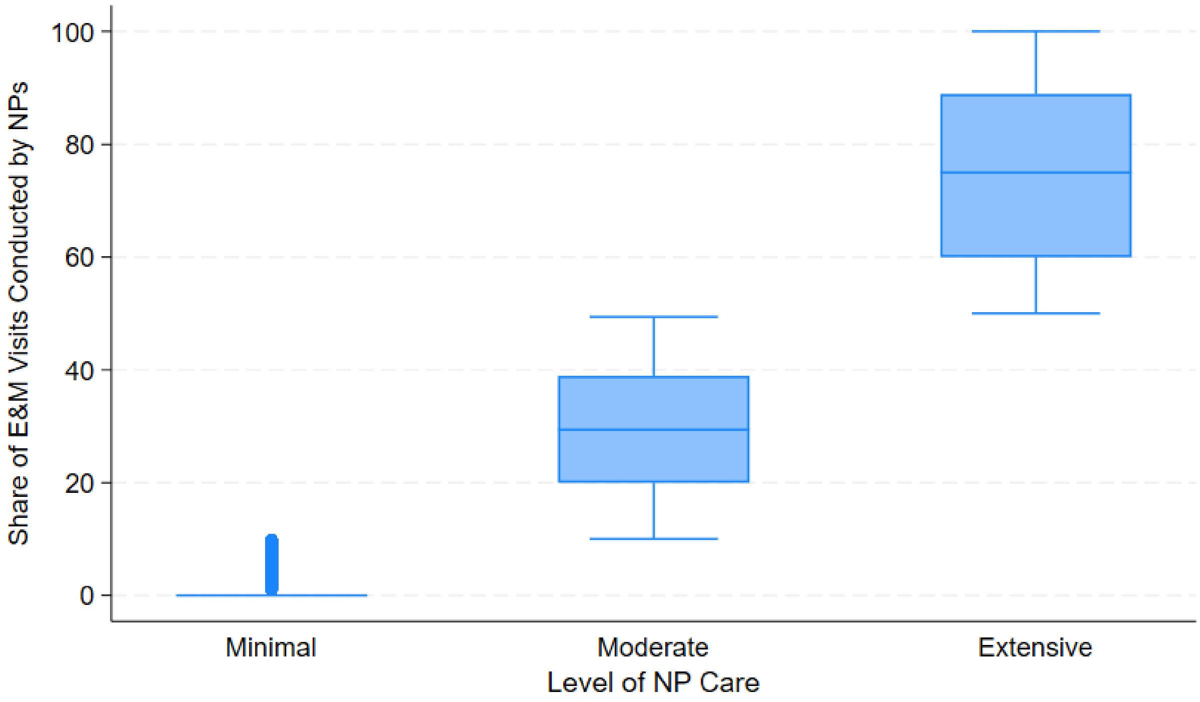

Note: E&M=evaluation and management, NP=nurse practitioner. See the Methods section (main text) for more details on the classification.

© 2024 Kosar CM et al. *JAMA Health Forum*.

eFigure 3. Scope of Practice Regulations by State at the Time of Study

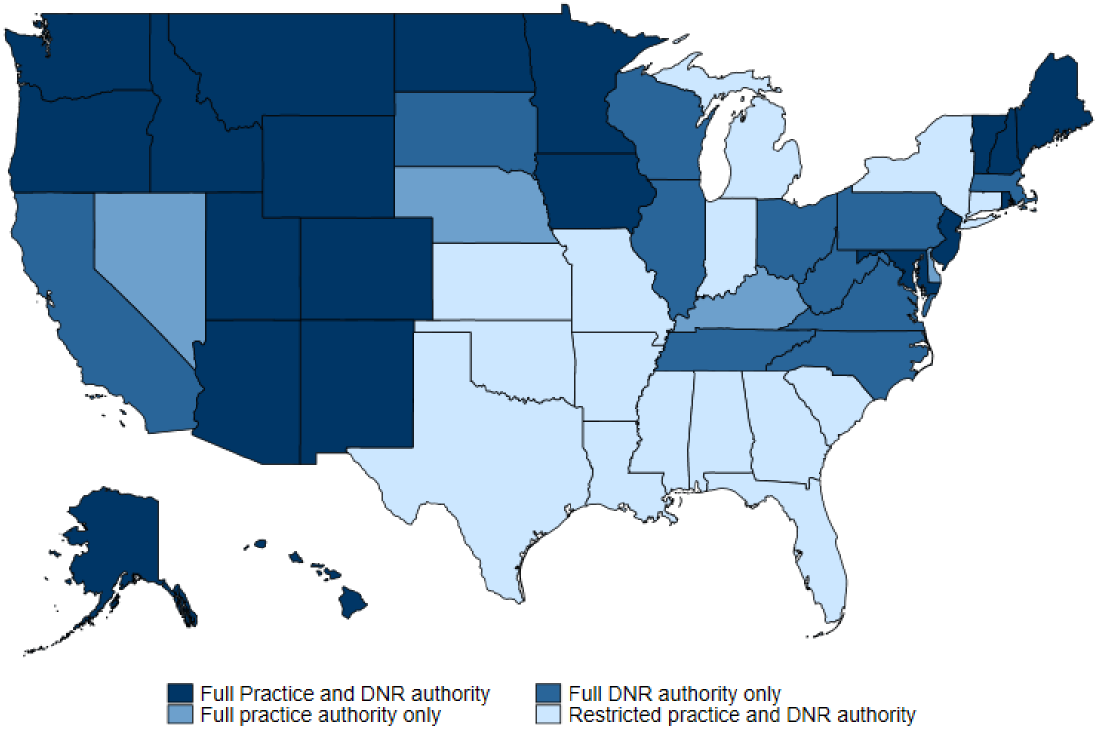

Note: DNR = Do Not Resuscitate. Scope of practice regulations were classified as of 2016. See the Methods section (main text) for more details on the classification.

© 2024 Kosar CM et al. *JAMA Health Forum*.

eTable 1. Crude and Adjusted Characteristics of Individuals in States with Restricted vs. Full Practice and Do Not Resuscitate Order Authority for Nurse Practitioners

	**Restricted vs Full DNR Authority States**				**Restricted vs Full Practice Authority States**			
	Not HRR-Adjusted		HRR-Adjusted[1]		Not HRR-Adjusted		HRR-Adjusted[1]	
Characteristic, (%)	Res.	Full	Res.	Full	Res.	Full	Res.	Full
Age at death, mean	86.3	87.0	86.5	86.7	86.5	87.0	86.5	87.0
Race/ethnicity, White	84.9	86.3	85.6	85.6	85.1	87.8	85.6	85.6
Race/ethnicity, Black	11.4	8.6	10.1	9.9	10.6	7.6	10.1	9.9
Race/ethnicity, Hispanic	1.6	3.4	2.5	2.5	2.4	3.0	2.5	2.5
Female sex	69.6	68.9	69.6	69.0	69.5	68.4	69.1	70.1
Years with dementia, mean	5.3	5.3	5.3	5.3	5.3	5.2	5.3	5.3
Dual Medicaid enrollment	77.1	73.3	77.0	73.4	76.6	69.0	76.6	69.2
No. baseline period E/M visits, mean	13.1	12.1	12.2	12.9	13.0	10.9	12.8	11.8
Share of E/M visits with PA billing, mean	5.1	5.2	4.9	5.4	5.1	5.1	5.3	4.6
Hypertension	98.0	96.7	97.3	97.4	97.6	96.2	97.2	97.8
Anemia	65.5	58.6	64.8	59.3	63.6	55.4	62.0	62.0
Atrial fibrillation	37.9	37.3	38.2	37.0	37.7	37.1	37.3	38.9
Cancer	20.8	21.1	21.2	20.7	20.9	21.2	20.7	21.8
Chronic kidney disease	71.7	68.6	71.4	68.9	71.1	66.0	70.3	69.5
Chronic obstructive pulmonary disease	32.1	27.6	31.1	28.6	30.7	26.3	30.4	27.4
Diabetes	60.5	55.4	59.2	56.7	59.0	53.3	58.0	57.8
Heart failure	55.3	50.1	54.2	51.2	53.8	47.9	52.7	52.7
Ischemic heart disease	80.7	76.7	79.3	78.0	79.4	75.5	78.7	78.6
Stroke	18.2	16.4	17.5	17.2	17.7	15.7	17.3	17.6
Drug use disorders	1.5	1.5	1.6	1.5	1.5	1.6	1.5	1.4
Peripheral vascular disease	54.9	57.2	56.8	55.3	56.3	54.8	56.4	54.5
Personality disorders	4.0	3.4	3.6	3.8	3.8	3.2	4.0	2.3
Pressure and chronic ulcers	34.0	31.0	32.6	32.3	33.2	29.1	32.5	32.1
Bipolar disorder	11.6	10.6	11.1	11.1	11.2	10.7	11.2	10.5
Depression	83.7	81.0	82.5	82.2	83.0	79.7	82.6	81.5
Schizophrenia	5.9	5.5	5.2	6.2	6.0	4.2	6.0	4.5
Population density, mean	1.9	1.3	1.5	1.7	1.7	1.2	1.6	1.6
Medicare Advantage penetration, mean	31.6	29.9	31.8	29.7	32.3	24.1	30.9	30.0
Social Deprivation Index, mean	50.7	44.5	48.2	47.0	49.1	41.0	47.7	47.1
Minimal NP care (<10% of visits)	39.7	41.3	41.4	39.6	40.5	40.7	41.6	35.9
Moderate NP care (10-50% of visits)	20.8	22.1	21.5	21.4	21.6	20.8	21.5	21.1
Extensive NP care (>50% of visits)	39.5	36.6	37.1	38.9	37.8	38.6	36.9	42.9
Nursing home star-rating, mean	3.2	3.3	3.3	3.2	3.2	3.4	3.2	3.4
Nursing home total beds, mean	138.0	135.1	136.9	136.1	137.9	130.3	137.9	130.4
% Medicaid of nursing home beds, mean	62.8	59.3	62.7	59.4	61.8	57.8	61.4	59.7
For-profit nursing home	69.6	69.9	65.6	70.1	69.4	63.6	67.0	73.6

Notes: HRR= Hospital Referral Region; DNR= Do Not Resuscitate; Res.=Restricted; No.= number of; E/M= Evaluation and Management; NP=nurse practitioner; PA=physician assistant. Population density is show as thousands of persons per square mile in the individual's county.

[1]To obtain HRR-adjusted estimates, we regressed each characteristic on state scope of practice authority status controlling for HRR fixed effects and then derived the adjusted mean the characteristic for individuals in states with restricted and expanded NP practice and DNR authority.

© 2024 Kosar CM et al. *JAMA Health Forum*.

eTable 2. Predicted Probability of End-of-Life Hospitalization by Level of Nurse Practitioner Care and Scope of Practice Regulation

	Practice Authority		DNR Authority	
Level of NP Care	Restricted	Full	Restricted	Full
Minimal (<10% of visits)	31.74	27.02	32.71	29.12
Moderate (10-50% of visits)	33.75	31.12	34.73	31.95
Extensive (>50% of visits)	33.24	29.82	34.06	31.03

Notes: NP=Nurse Practitioner. Estimates are adjusted mean predictions from a regression of end-of-life hospitalization on all study covariates without NP care level or scope of practice regulation included. See the Methods section (main text) for more details on the model specification and variable definitions.

© 2024 Kosar CM et al. *JAMA Health Forum*.

eTable 3. Association Between Nurse Practitioner Care Level and End-of-life Outcomes Among Nursing Home Residents with Dementia

	Hospitalization			Hospice Enrollment		
Level of NP Care	Adj. Mean (95% CI)	*β* (95% CI)	*p*	Adj. Mean (95% CI)	*β* (95% CI)	*p*
Minimal (<10% of visits)	32.31 (32.06, 32.55)	Referent	-- --	53.57 (53.30, 53.84)	Referent	-- --
Moderate (10-50% of visits)	31.64 (31.32, 31.96)	-0.67 (-1.08, -0.26)	0.001	55.47 (55.12, 55.83)	1.90 (1.45, 2.31)	<0.001
Extensive (>50% of visits)	31.62 (31.37, 31.87)	-0.69 (-1.06, -0.32)	<0.001	55.57 (55.29, 55.85)	2.00 (1.58, 2.11)	<0.001

Notes: NP=Nurse Practitioner, Adj.= Adjusted; CI= Confidence Interval. Estimates were multiplied by 100 to facilitate interpretation as percentage points. The regression model included hospital referral region fixed effects. See the Methods section (main text) for more details on the model specification and variable definitions.

© 2024 Kosar CM et al. *JAMA Health Forum*.

eTable 4. Adjusted End-of-Life Hospitalization Rates in States with Restricted vs Full Practice and Do Not Resuscitate Order Authority for Nurse Practitioners

(A) Practice Authority

	States with Restricted Authority			States with Full Authority		
Level of NP Care	Adj. Mean (95% CI)	β (95% CI)	*p*	Adj. Mean (95% CI)	β (95% CI)	*p*
Minimal (<10% of visits)	32.03 (31.70, 32.37)	Referent		33.47 (32.45, 34.48)	Referent	
Moderate (10-50% of visits)	31.53 (31.12, 31.94)	-0.51 (-0.96, -0.05)	0.03	32.10 (30.96, 33.25)	-1.36 (-2.28, -0.45)	0.004
Extensive (>50% of visits)	31.61 (31.26, 31.96)	-0.43 (-0.84, -0.01)	0.04	31.70 (30.68, 32.72)	-1.76 (-2.52, -1.00)	<0.001

(B) DNR Authority

	States with Restricted Authority			States with Full Authority		
Level of NP Care	Adj. Mean (95% CI)	β (95% CI)	*p*	Adj. Mean (95% CI)	β (95% CI)	*p*
Minimal (<10% of visits)	32.79 (32.16, 33.42)	Referent		31.84 (31.24, 32.45)	Referent	
Moderate (10-50% of visits)	32.11 (31.41, 32.82)	-0.67 (-1.27, -0.07)	0.02	31.16 (30.49, 31.83)	-0.69 (-1.24, -0.14)	0.01
Extensive (>50% of visits)	32.71 (32.08, 33.34)	-0.08 (-0.60, 0.44)	0.77	30.54 (29.93, 31.15)	-1.80 (-1.70, -0.81)	<0.001

Notes: DNR=Do Not Resuscitate; NP=Nurse Practitioner; Adj.= Adjusted; CI= Confidence Interval. Estimates were derived from a regression of end-of-life hospitalization onto level of NP care interacted with scope of practice authority status. Practice and DNR authority were included in separate regressions. Regression models included hospital referral region fixed effects. See the Methods section (main text) for more details on the model specification and variable definitions.

© 2024 Kosar CM et al. *JAMA Health Forum*.

eTable 5. Association Between Nurse Practitioner Care Level, Scope of Practice Regulation, and their Interaction with End-of-life Hospitalizations

	(1) Practice Authority			(2) DNR Authority		
Variable	*β*	95% CI	*p*	*β*	95% CI	*p*
Minimal NP care (<10% of visits)	Referent	-- --	-- --	Referent	-- --	-- --
Moderate NP care (10-50% of visits)	-0.51	-0.96, -0.05	0.029	-0.67	-1.27, -0.07	0.028
Extensive NP care (>50% of visits)	-0.43	-0.84, -0.01	0.043	-0.08	-0.60, 0.44	0.770
Full authority[1]	1.43	0.22, 2.65	0.021	-0.94	-2.07, 0.18	0.101
Moderate NP care*Full authority	-0.86	-1.87, 0.16	0.099	0.01	-0.82, 0.79	0.973
Extensive NP care*Full authority	-1.34	-2.19, -0.49	0.002	-1.23	-1.93, -0.53	0.001

Notes: DNR=Do Not Resuscitate; CI= Confidence Interval; NP=Nurse Practitioner; Estimates were multiplied by 100 to facilitate interpretation as percentage point differences in outcomes between individuals with different NP care levels and across states with and without full practice and DNR authority for NPs. Practice and DNR authority were included in separate regressions. Regression models included hospital referral region fixed effects. See the Methods section (main text) for more details on the model specification and variable definitions.

[1]Here, the coefficient on full authority can be interpreted as the percentage point difference in the probability of hospitalization enrollment between those receiving minimal NP care in states with full authority vs restricted authority.

© 2024 Kosar CM et al. *JAMA Health Forum*.

eTable 6. Adjusted End-of-Life Hospice Enrollment Rates in States with Restricted vs Full Practice and Do Not Resuscitate Order Authority for Nurse Practitioners

**(A) Practice Authority**	States with Restricted Authority			States with Full Authority		
Level of NP Care	Adj. Mean (95% CI)	β (95% CI)	*p*	Adj. Mean (95% CI)	β (95% CI)	*p*
Minimal (<10% of visits)	53.52 (53.14, 53.89)	Referent		53.81 (52.65, 54.98)	Referent	
Moderate (10-50% of visits)	55.05 (54.60, 55.51)	1.53 (1.04, 2.03)	<0.001	57.32 (56.01, 58.63)	3.51 (2.45, 4.56)	<0.001
Extensive (>50% of visits)	55.29 (54.90, 55.68)	1.77 (1.32, 2.23)	<0.001	56.69 (55.53, 57.86)	2.88 (1.99, 3.77)	<0.001
**(B) DNR Authority**	States with Restricted Authority			States with Full Authority		
Level of NP Care	Adj. Mean (95% CI)	β (95% CI)	*p*	Adj. Mean (95% CI)	β (95% CI)	*p*
Minimal (<10% of visits)	52.54 (51.86, 53.22)	Referent		54.56 (53.89, 55.23)	Referent	
Moderate (10-50% of visits)	53.37 (52.61, 54.13)	0.83 (0.19, 1.46)	0.01	57.48 (56.74, 58.23)	2.92 (2.29, 3.56)	<0.001
Extensive (>50% of visits)	53.91 (53.23, 54.59)	1.37 (0.81, 1.93)	<0.001	57.18 (56.51, 57.86)	2.62 (2.04, 3.20)	<0.001

Notes: DNR=Do Not Resuscitate; NP=Nurse Practitioner; Adj.= Adjusted; CI= Confidence Interval. Estimates were derived from a regression of end-of-life hospice enrollment onto level of NP care interacted with scope of practice authority status. Practice and DNR authority were included in separate regressions. Regression models included hospital referral region fixed effects. See the Methods section (main text) for more details on the model specification and variable definitions.

© 2024 Kosar CM et al. *JAMA Health Forum*.

eTable 7. Association Between Nurse Practitioner Care Level, Scope of Practice Regulation, and Their Interaction with End-of-life Hospice Enrollment

	(1) Practice Authority			(2) DNR Authority		
Variable	*β*	95% CI	*p*	*β*	95% CI	*p*
Minimal NP care (<10% of visits)	Referent	-- --	-- --	Referent	-- --	-- --
Moderate NP care (10-50% of visits)	1.53	1.04, 2.03	<0.001	0.83	0.19, 1.46	0.01
Extensive NP care (>50% of visits)	1.77	1.32, 2.23	<0.001	1.37	0.81, 1.93	<0.001
Full authority[1]	0.30	-1.10, 1.69	0.677	2.02	0.78, 3.23	0.001
Moderate NP care*Full authority	1.97	0.81, 3.14	0.001	2.10	1.21, 2.98	<0.001
Extensive NP care*Full authority	1.11	0.12, 2.09	0.027	1.25	0.47, 2.03	0.002

Notes: DNR=Do Not Resuscitate; CI= Confidence Interval; NP=Nurse Practitioner; Estimates were multiplied by 100 to facilitate interpretation as percentage point differences in outcomes between individuals with different NP care levels and across states with and without full practice and DNR authority for NPs. Practice and DNR authority were included in separate regressions. Regression models included hospital referral region fixed effects. See the Methods section (main text) for more details on the model specification and variable definitions.

[1]Here, the coefficient on full authority can be interpreted as the percentage point difference in the probability of hospice enrollment between those receiving minimal NP care in states with full authority vs restricted authority.

© 2024 Kosar CM et al. *JAMA Health Forum*.

eTable 8. Adjusted End-of-Life Hospitalization Rates by Nurse Practitioner Level Care Level and Scope of Practice Regulation Among Residents with High Visit Volume

**(A) Practice Authority**						
	States with Restricted Authority			States with Full Authority		
Level of NP Care	Adj. Mean (95% CI)	*β* (95% CI)	*p*	Adj. Mean (95% CI)	*β* (95% CI)	*p*
Minimal (<10% of visits)	32.52 (32.07, 32.98)	Referent		34.14 (32.61, 35.67)	Referent	
Moderate (10-50% of visits)	32.49 (32.01, 32.96)	-0.03 (-0.60, 0.52)	0.89	33.03 (31.47, 34.59)	-1.11 (-2.36, 0.13)	0.08
Extensive (>50% of visits)	32.43 (32.02, 32.84)	-0.09 (-0.62, 0.44)	0.74	32.85 (31.41, 34.29)	-1.29 (-2.42, -0.16)	0.03
**(B) DNR Authority**						
	States with Restricted Authority			States with Full Authority		
Level of NP Care	Adj. Mean (95% CI)	*β* (95% CI)	*p*	Adj. Mean (95% CI)	*β* (95% CI)	*p*
Minimal (<10% of visits)	33.33 (32.47, 34.19)	Referent		32.22 (31.38, 33.06)	Referent	
Moderate (10-50% of visits)	33.28 (32.39, 34.16)	-0.05 (-0.80, 0.07)	0.89	31.84 (30.99, 32.69)	-0.38 (-1.07, 0.32)	0.29
Extensive (>50% of visits)	33.81 (33.00, 34.62)	0.48 (-0.22, 1.16)	0.17	31.19 (30.40, 31.98)	-1.03 (-1.69, -0.37)	<0.01

Notes: DNR=Do Not Resuscitate; NP=Nurse Practitioner; Adj.= Adjusted; CI= Confidence Interval. Residents with 8 or more evaluation and management visits were considered 'high-volume.' Estimates were derived from a regression of end-of-life hospitalization onto level of NP care interacted with scope of practice authority status. Practice and DNR authority were included in separate regressions. Regression models included hospital referral region fixed effects. See the Methods section (main text) for more details on the model specification and variable definitions.

© 2024 Kosar CM et al. *JAMA Health Forum*.

eTable 9. Adjusted End-of-Life Hospice Enrollment Rates by Nurse Practitioner Level Care Level and Scope of Practice Regulation Among Residents with High Visit Volume

**(A) Practice Authority**						
	States with Restricted Authority			States with Full Authority		
Level of NP Care	Adj. Mean (95% CI)	β (95% CI)	*p*	Adj. Mean (95% CI)	β (95% CI)	*p*
Minimal (<10% of visits)	55.06 (54.56, 55.57)	Referent		54.87 (53.10, 56.63)	Referent	
Moderate (10-50% of visits)	56.36 (55.83, 56.88)	1.29 (0.69, 1.90)	<0.001	57.51 (55.72, 59.29)	2.64 (1.21, 4.06)	<0.001
Extensive (>50% of visits)	56.76 (56.31, 57.22)	1.69 (1.12, 2.28)	<0.001	57.03 (55.39, 58.68)	2.17 (0.86, 3.47)	<0.01
**(B) DNR Authority**						
	States with Restricted Authority			States with Full Authority		
Level of NP Care	Adj. Mean (95% CI)	β (95% CI)	*p*	Adj. Mean (95% CI)	β (95% CI)	*p*
Minimal (<10% of visits)	53.69 (52.76, 54.62)	Referent		54.56 (53.89, 55.23)	Referent	
Moderate (10-50% of visits)	54.27 (53.32, 55.22)	0.58 (0.20, 1.35)	0.15	57.48 (56.74, 58.23)	2.41 (1.61, 2.20)	<0.001
Extensive (>50% of visits)	54.59 (53.71, 55.46)	0.89 (0.16, 1.62)	0.02	57.18 (56.51, 57.86)	2.63 (1.87, 3.38)	<0.001

Notes: DNR=Do Not Resuscitate; NP=Nurse Practitioner; Adj.= Adjusted; CI= Confidence Interval. Residents with 8 or more evaluation and management visits were considered 'high-volume.' Estimates were derived from a regression of end-of-life hospice enrollment onto level of NP care interacted with scope of practice authority status. Practice and DNR authority were included in separate regressions. Regression models included hospital referral region fixed effects. See the Methods section (main text) for more details on the model specification and variable definitions.

© 2024 Kosar CM et al. *JAMA Health Forum*.
