# Supplementary material for: Nurse Practitioner Care, Scope of Practice, and End-of-Life Outcomes for Nursing Home Residents With Dementia
Source: JAMA Health Forum. 2024 May 10;5(5):e240825. doi: 10.1001/jamahealthforum.2024.0825 (PMC11087831; doi:10.1001/jamahealthforum.2024.0825)
Supplement: Supplement 2. — Data Sharing Statement [file jamahealthforum-e240825-s002.pdf]

Data Sharing Statement

Data
Data available: No

Additional Information
Explanation for why data not available: We cannot share study data due to the terms of our data use agreement however, statistical code can be made available upon request.
